# Hybrid Assembly and Annotation of the Genome of the Indian *Punica granatum*, a Superfood

**DOI:** 10.3389/fgene.2022.786825

**Published:** 2022-05-11

**Authors:** Talambedu Usha, Sushil Kumar Middha, Dinesh Babu, Arvind Kumar Goyal, Anupam J. Das, Deepti Saini, Aditya Sarangi, Venkatesh Krishnamurthy, Mothukapalli Krishnareddy Prasannakumar, Deepak Kumar Saini, Kora Rudraiah Sidhalinghamurthy

**Affiliations:** ^1^ Department of Biochemistry, Bangalore University, Bengaluru, India; ^2^ DBT-BIF Facility, Department of Biotechnology, Maharani Lakshmi Ammanni College for Women, Bengaluru, India; ^3^ Faculty of Pharmacy and Pharmaceutical Sciences, University of Alberta, Edmonton, AB, Canada; ^4^ Centre for Bamboo Studies, Department of Biotechnology, Bodoland University, Kokrajhar, India; ^5^ Molsys Pvt. Ltd., Bengaluru, India; ^6^ Protein Design Private Limited, Bengaluru, India; ^7^ Basesolve Informatics Private Limited, Ahmedabad, India; ^8^ Genotypic Technology Pvt Limited, Bengaluru, India; ^9^ Department of Plant Pathology, University of Agricultural Sciences, Bengaluru, India; ^10^ Department of Molecular Reproduction Development and Genetics, Indian Institute of Science, Bengaluru, India

**Keywords:** Punica granatum (cultivar Bhagwa), flavonoids biosynthesis, phenylpropanoid pathway, whole genome, oxford nanopore, hybrid assembly, next-generation sequencing

## Abstract

The wonder fruit pomegranate (*Punica granatum*, family Lythraceae) is one of India’s economically important fruit crops that can grow in different agro-climatic conditions ranging from tropical to temperate regions. This study reports high-quality *de novo* draft hybrid genome assembly of diploid Punica cultivar “Bhagwa” and identifies its genomic features. This cultivar is most common among the farmers due to its high sustainability, glossy red color, soft seed, and nutraceutical properties with high market value. The draft genome assembly is about 361.76 Mb (N50 = 40 Mb), ∼9.0 Mb more than the genome size estimated by flow cytometry. The genome is 90.9% complete, and only 26.68% of the genome is occupied by transposable elements and has a relative abundance of 369.93 SSRs/Mb of the genome. A total of 30,803 proteins and their putative functions were predicted. Comparative whole-genome analysis revealed *Eucalyptus grandis* as the nearest neighbor. KEGG-KASS annotations indicated an abundance of genes involved in the biosynthesis of flavonoids, phenylpropanoids, and secondary metabolites, which are responsible for various medicinal properties of pomegranate, including anticancer, antihyperglycemic, antioxidant, and anti-inflammatory activities. The genome and gene annotations provide new insights into the pharmacological properties of the secondary metabolites synthesized in pomegranate. They will also serve as a valuable resource in mining biosynthetic pathways for key metabolites, novel genes, and variations associated with disease resistance, which can facilitate the breeding of new varieties with high yield and superior quality.

## Introduction

*Punica granatum* L (family: Lythraceae), alias pomegranate, is one of the ancient and well-known edible fruits and is well known in Ayurveda as a Rasayana (life- and health-span enhancing agent) (Balasubramani et al., 2014). The genus *Punica* (Angiosperm Phylogeny Group IV classification) contains only two sister species with a classic intercontinental disjunction dispersion, one in Western Asia, Iran (*P. granatum*), and the other in Socotra Island, Yemen (*P. protopunica*) (Kandylis and Kokkinomagoulos, 2020). *P. granatum* is culturally considered a symbol of fertility, abundance, blessings, immortality, and invincibility because of its pharmaceutical and nutraceutical values (Usha et al., 2020). The plant is domesticated in Asia, the Middle East, Southern Europe, the United States, and the milder climatic regions of Africa for food, religious, and medicinal uses. Interestingly, every part of the pomegranate, namely, the fruit, rind, flowers, leaves, roots, and wood, has therapeutic and economic values (Medjakovic and Jungbauer, 2013). The chemical constituents of the fruits vary based on the cultivar, growth climate, maturity time, cultivation method, and storage conditions (Fadavi et al., 2005). The peel, seed, bark, leaves, seed oil, juice, and heartwood of pomegranate contain several potentially active phytochemicals such as alkaloids, anthocyanins, flavonoids, gallotannins, organic acids, polyphenols, proanthocyanidins, tannins, terpenes, tocopherols, conjugated linolenic acids, triacylglycerols, sterols, steroids, minerals, and complex polysaccharides (Sreekumar et al., 2014; Usha et al., 2020). There is ample evidence demonstrating the therapeutic effects of pomegranate and its derived products in arthritis, bacterial infections, diabetes, dental conditions, cardiac disorders, erectile dysfunction, hyperlipidemia, Alzheimer’s, infant brain ischemia, obesity (Jurenka, 2008), cancer (breast, skin, prostate, colon, thyroid, and osteosarcoma) (Usha et al., 2020), AIDS (Sreekumar et al., 2014), and inflammation (Vučić et al., 2005). Moreover, 73 clinical trials have been conducted to date, exploring the efficacy and safety of pomegranate mono- and polyherbal medicines in a wide array of ailments^1^.

Pomegranate is thus categorized as a superfood, and there is a soaring demand for its fruit, processed products, and byproducts. In addition to its therapeutic value, the unique morphological characteristics, namely, 1) andromonoecy (Lazare et al., 2020), 2) each aril being derived from a single ovule accompanied by independent fertilization, and 3) edible juicy, fleshy external seed coat encapsulating the inner fibrous seed coat (Qin et al., 2017), make *P. granatum* a fascinating fruit to study reproduction, selective adaptation, and evolution in plants.

The pomegranate plant is unique in that it can thrive in various agro-climatic conditions, from tropical to temperate, which are typically deemed unsuitable for cultivating many other economically and medicinally important fruits (Chandra et al., 2010). Due to its high therapeutic value and global demand, there has been a tremendous increase in export potential in recent years, with notably India being one of the largest exporters of pomegranate to the world. Of the ten available cultivars in India, “Bhagwa” is the sustained variety that is primarily exported and the most popular among the farmers (Chandra et al., 2010). Thus, there is a compelling need to use modern molecular genetics methods to obtain insights into this cultivar’s genetic and molecular features, aimed at producing high-quality pomegranate fruits with an attractive appearance and a relatively high content of health-promoting ingredients, and disease resistance. Three currently available genome sequences of the Chinese varieties “Dabenzi” (328.38 Mb) (Qin et al., 2017), “Taishanhong” (274 Mb) (Yuan et al., 2018), and “Tunisia” (320.31 Mb) (Luo et al., 2020) and their gene annotations have facilitated advances in basic research, comparative, and evolutionary genomics studies of *P. granatum.* While these resources help dissect the metabolic features, the sequence for the common Indian cultivar must be obtained for trait improvement and to further enhance the production of secondary metabolites in the Indian variety “Bhagwa.”

The current study presents the first *de novo* draft genome, hybrid assembly of the *P. granatum* of the Indian soft-seeded variety “Bhagwa” by Illumina and Oxford Nanopore sequencing technologies. We have identified a large set of genes involved in the production of secondary metabolites with medicinal values, such as phenylpropanoids, flavonoids, and tocopherols. The Hidden Gene prediction model unraveled the similarity of the *P. granatum* genome to the *Eucalyptus grandis.* The repeat elements and microsatellites in the assembled genome occupied only 26.68% and 0.06% of the genome. Our new findings of this draft genome will help understand the metabolic traits and improve the quality of the “Bhagwa” variety and facilitate approaches for increasing the content of secondary metabolites. This first draft whole-genome sequence of an Indian cultivar also presents an essential template for comparative genome analysis for crops from different geographic regions.

## Materials and Methods

**FIGURE 1:**
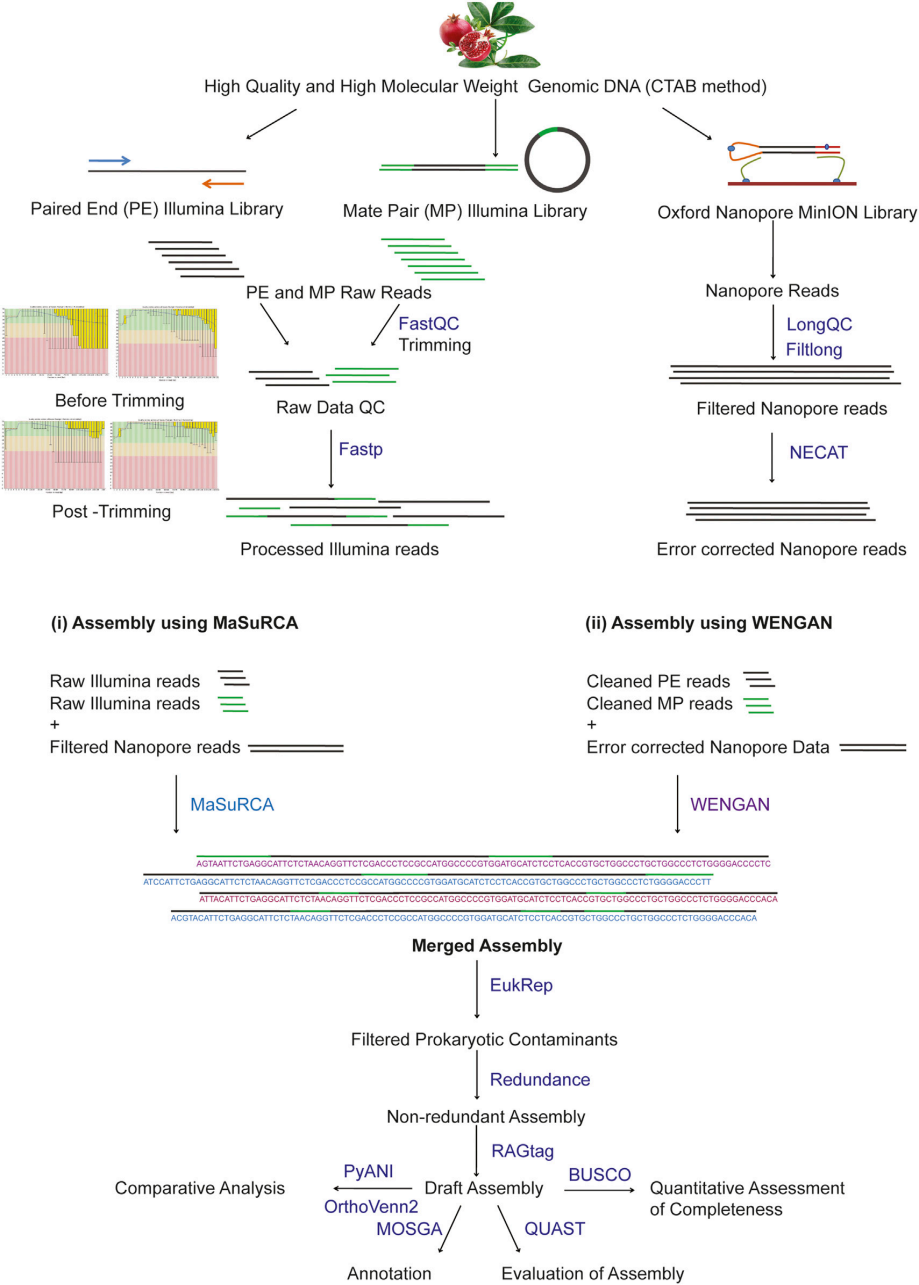
Overview of the study from the isolation of DNA to assembly and analysis of the genome.

The complete workflow adopted in the study is provided in Figure 1.

### Estimation of Nuclear DNA Content of the Leaf From *Punica granatum* “Bhagwa” Variety by Flow Cytometry

Approximately 1 cm^2^ of young *P. granatum* leaf tissue was taken and nuclear suspensions were made according to Galbraith et al. (1983), with slight changes. The leaves of a sample were chopped with a razor blade in a Petri dish with 1 ml cold Galbraith nuclear isolation buffer: 45 mM magnesium chloride (Merck, Germany), 30 mM tri-sodium citrate, 20 mM 4-morpholinepropanesulfonic acid, 3-propanesulfonic acid (MOPS) pH 7, 10 mM sodium metabisulfite, 1% polyvinylpyrrolidone 10,000, 1% (w/v), and Triton X-100 (Sigma-Aldrich, United States). The nuclear suspension was filtered using a nylon mesh of 50 µm to eliminate cell fragments and large debris. Nuclei were stained with 50 μg ml^−1^ propidium iodide (PI) (Sigma-Aldrich, United States), a DNA-intercalating fluorochrome, and 50 μg ml^−1^ RNase A (Sigma, United States) was also added. The samples were then incubated in the dark for 15 min on ice before flow cytometric evaluation. Chicken erythrocyte nuclei were used as a standard for nuclear content estimation. The stained standard and plant nuclei (5,000–10,000 events) were then analyzed on a BD Accuri C6 flow cytometer at 488 nm, and the fluorescence signal was collected using a 585/40 nm bandpass filter.

### Isolation of Genomic DNA From the Leaves of the *Punica granatum* “Bhagwa” Variety

The fresh leaves from a sapling of *P. granatum*, collected from Gandhi Krishi Vigyana Kendra (GKVK), Bengaluru, India, were harvested for genome sequencing. “Bhagwa” was chosen for genome sequencing due to its extensive domestication in India. The fresh leaves were frozen immediately in liquid nitrogen to extract high molecular weight genomic DNA using the cetyltrimethylammonium bromide (CTAB) method. 1 gm of *P. granatum* leaves were weighed and frozen in liquid nitrogen and homogenized at 4,000 rpm for 10 s, using TOMY Micro Smash MS 100 cell disruptor. The homogenization step was repeated twice; the thus obtained lysate was mixed with 1 ml CTAB lysis buffer (100 mM Tris base, 2% CTAB, 1.5 M NaCl, 20 mM EDTA, 1% sodium dodecyl sulphate (SDS), and 1% polyethylene glycol (Sigma, United States) and incubated for 30 min at 65°C. 20 µl of proteinase K solution was added to the vial and incubated for 1 h at 56°C.

Further, proteinase K (Sigma, United States) activity was arrested by incubating at 65°C for 10 min. The vials were centrifuged at 8,000 rpm for 10 min at RT to remove the debris. 20 µl of RNAase (20 mg/ml; Sigma, United States) was added to the collected supernatant and incubated at 65°C for 10 min. To the supernatant, an equal volume of phenol: chloroform: isoamyl alcohol (25:24:1) (Merck, Germany) mixture was added and mixed gently. The mixture was centrifuged at 13,200 rpm for 10 min at 4°C to separate the phases. The aqueous phase from the centrifuged vials was transferred to a fresh vial, and an equal volume of 100% isoamyl-alcohol was added and incubated at −80°C for 1 h. The vials were left at room temperature for 5 min and centrifuged at 13,200 rpm for 20 min at 4°C. The pellet was washed twice with 70% ethanol. The extracted DNA was run on 1% agarose gel to assess the quality. DNA was also analyzed for its concentration and purity using NanoDrop™ Spectrophotometer and Qubit 4 Fluorometer (Thermo Fisher Scientific, Massachusetts, United States). 1x TE buffer was added to the pellets of the two vials for Oxford Nanopore library preparation. The pellets in the other two vials were resuspended in ×10 Tris buffer (pH: 8) and were further used for Illumina library preparation.

### Library Preparation Methods and Whole-Genome Sequencing

The extracted DNA was purified using Qiagen DNeasy Blood & Tissue kit column (Qiagen, Germany). Paired-end (PE) and mate-pair (MP) sequencing libraries with insert sizes of 400–550 bp and 300 bp to 1,000 bp, respectively, were constructed and sequenced on the Illumina HiSeq 2,000 platform to obtain low error short reads. For nanopore sequencing, 2 µg of genomic DNA was end-repaired (NEBNext Ultra II End Repair Kit, New England Biolabs, MA, United States) and cleaned with ×1 AMPure beads (Beckman Coulter, United States). NEB blunt/TA ligase (New England Biolabs, MA, United States) was used to perform adapter ligations (AMX) for 30 min. Library mix was cleaned up using 0.4× AMPure beads (Beckman Coulter, United States) and finally eluted in 16 µl of elution buffer. A total of 480 ng of sequencing library was obtained and used for sequencing. Long reads were obtained by sequencing on MinION MklB (Oxford Nanopore Technologies, Oxford, United Kingdom) using spot on flow cell (R9.4), and base calling was performed using Metrichor Nanopore. A total of 46.2 Gb of raw data was generated on the Oxford Nanopore and Illumina platforms.

### Raw Data Processing and *In Silico* Genome Size Estimation

The Illumina paired-end and mate-pair raw reads were checked for quality using FastQC (Andrews, 2010). FASTP (Chen et al., 2018) was used to process Illumina raw reads for adapters and trim low-quality bases. The raw nanopore reads were processed, and the quality was checked by LongQC (Fukasawa et al., 2020), followed by quality trimming using Filtlong^2^. The processed nanopore reads were corrected using NECAT (Chen et al., 2021). The genome size estimation of *P. granatum* was carried out using GenomeScope V2.0. (Vurture et al., 2017).

### *De Novo* Hybrid Assembly of the Nuclear Genome of *P. granatum*

The *P. granatum* draft genome was assembled using MaSuRCA V.3.4.2^3^ (Zimin et al., 2013) and WENGAN (Di Genova et al., 2021) hybrid assemblers individually based on the Illumina paired-end reads and corrected nanopore reads. The draft assemblies generated by MaSuRCA and WENGAN were merged. Prokaryotic contamination was removed from the merged assembly using EukRep (West et al., 2018), followed by developing one non-redundant set of contigs by assembly reduction using Redundans (Pryszcz and Gabaldón, 2016). The reduced contigs were further scaffolded based on *P. granatum* reference (GCF_007655135.1) using RagTag (Alonge et al., 2019).

### Qualitative Analysis of *De Novo P. granatum* Draft Genome Assembly

Benchmarking set of Universal Single-Copy Orthologues (BUSCO-version 5) (Simão et al., 2015) was used for the identification of single-copy orthologs against eudicot odb10 lineage in the *P. granatum* draft assembly and compared with *P. granatum* cultivarDabenzi [GCA_002201585.1], Taishanhong [GCA_002864125.1], isolate Tunisia 2019 [GCF_007655135.1], and strain AG 2017 [GCA_002837095.1]. BUSCO robustly estimates the completeness of the genome, and BUSCOs are conserved single-copy orthologs that are predicted to be present in the complete genome. Therefore, the number of fragmented, duplicated, present, and missing BUSCOs can be used in the quality control of the assembled genome.

### Nuclear Genome Annotations

#### Identification of Transposable Elements

Transposable elements (TEs) are major players in the structure and evolution of plant genomes. Their ability to locomote around and replicate within genomes are probably the most essential contributors to genome size and plasticity (Sahebi et al., 2018). Thorough annotation of TEs is ideal for dealing with the deluge of genome data. Therefore, *de novo* repeat identification was performed by Repeat Modeller2 (Flynn et al., 2020), LongRepMarker (Liao et al., 2021), and Extensive De Novo TE Annotator (EDTA) (Ou et al., 2019). The unclassified repeats were further classified using DeepTE (Yan et al., 2020). The *de novo* repeats identified by all the above three methods were merged and clustered using cd-hit-est with a 99% threshold to generate one non-redundant repeat library. The draft genome assembly was used to unravel the transpositional landscape of *P. granatum* using RepeatMasker 4.0.7^4^ against the *de novo* constructed repeat library.

#### Prediction of Microsatellites

The plant genomes are filled with low complexity sequences such as simple sequence repeats (SSRs). The MISA tool^5^ was used to screen for the presence of SSRs in scaffolds. The sequences were annotated and screened for the most frequent type of SSR motif families and mono-repeats recurring a minimum of 10 times, di-repeats recurring a minimum of 5 times, and tri/tetra/penta/hexa-repeats recurring a minimum of 5 times. SSR statistics were generated by PySSRstat^6^.

#### Gene Annotations

Genome annotation was done using Modular Open Source Genome Annotator MOSGA (Martin et al., 2021). BRAKER2 (Brůna et al., 2021) was used for gene prediction, with orthology-based-evidence mode using OrthoDB as a data source that relies on GeneMark-EP spaln and DIAMOND (Buchfink et al., 2015). Prediction of tRNA sequences was performed using tRNAscan-SE2.0 (Chan et al., 2021). Prediction of rRNAs was done using Barrnap^7^. Functional gene prediction was made by comparison against Swiss-Prot and EggNog 5 protein databases. The gene model was obtained in GFF format. The transcript sequences were extracted from the GFF file using the gffread utility^8^. The transcript FASTA sequences were subjected to blastx against the Eudicotyledons (taxonomy id: 22663) of NCBI non-redundant database using DIAMOND [parameters: -max-target-seqs 20 --outfmt 5 --sensitive -e 1e-5 -b12 -c1 --taxonlist 22,663]. The BLASTX outputs in XML format were further annotated using BLAST2GO (Conesa et al., 2005).

#### Comparative Genomic Analysis Between Pomegranate and Other Plant Species

A gene family cluster analysis of the complete gene sets of pomegranate (*P. granatum*), *E. grandis*, apple (*Malus domestica*), arabidopsis (*Arabidopsis thaliana*), and grape (*Vitis vinifera*) was performed using OrthoVenn2 (https://orthovenn2.bioinfotoolkits.net/).

#### Phylogenetic Tree Based on Average Nucleotide Identity Analysis

We calculated the average nucleotide identity between the *P. granatum* draft genome with *Prunus* to check the genetic relatednes*s. persica* (cultivar_Lovell), *P. granatum* (cultivar Bhagwa, Dabenzi, Taishanhong, Tunisia 2019, and strain AG 2017), *Sonneratia. alba*, *Vitis. vinifera* (cultivar PN40024), *Sonneratia. caseolaris*, *Eucalyptus. grandis* (isolate ANBG69807), *Corymbia. maculata* (isolate sf003), *Angophora. floribunda* (isolate sf002), and *Corymbia citriodora* (subsp. variegata) on the “pyANI” software.

#### Identification and Analysis of the Variants in *P. granatum* Genome

The trimmed Illumina paired-end reads were aligned using bowtie2^9^ against the representative genome reference of *P. granatum* obtained from NCBI (GCF_007655135.1_ASM765513v2). The aligned reads were sorted and indexed using Samtools (Li et al., 2009). The aligned reads were utilized in variant calling using Genome Analysis Toolkit (GATK) (DePristo et al., 2011) with optimal practices for germline SNPs and indel calling^10^ (Pramesh et al., 2020). At first, the HaplotypeCaller was called without Base Quality Score Recalibration (BQSR), the thus obtained variants were fed to BQSR as know variants, and a recalibration was achieved, while no other modifications were made in the workflow. A set of hard filters such as “QD < 2.0 || FS > 200.0 || ReadPosRankSum < −20.0 || InbreedingCoeff < −0.8”, “SB >= 0.10 || QD < 5.0 || HRun >= 4” and other parameters were applied, such as cluster size 3, mask extension 5, and cluster window size 10. Thus, the filtered variants were predicted using snpEFF (Cingolani et al., 2012) with a custom-built effects database (http://snpeff.sourceforge.net/SnpEff_manual.html#databases) for *P. granatum.*

## Results

### Sequence Assembly

Flow cytometric analysis projected the *P. granatum* genome to be around 352.38 Mb (Supplementary Figure S1). The genome was sequenced using two sequencing platforms: Illumina (short-read technology) and Oxford Nanopore (long-read technology). We generated 52 Gb paired reads (2*150 bp), 11 GB mate-pair reads, and 2 Gb long reads of 2.31956 Kb average length. A total of 65 Gb of data representing ×155 fold coverage was generated (Supplementary Table S1). Data obtained through Illumina sequencing technology was also used to estimate the genome size and was found to be 276.60 Mb. (Supplementary Figure S2).

The raw reads (Bioproject: PRJNA407279) were filtered based on the quality score, trimmed (Supplementary Table S1), and then assembled into contigs using assemblers MaSuRCA V3.4.2 and WENGAN. The aforementioned assembler statistics resulted in 122 contigs of maximum length up to 88134655 bp with an N50 of 40 Mb, L50 of 3, and a hybrid assembly of 361.76 Mb of *P. granatum* (Table 1). The assembled genome was made up of 38.86% of GC.

**TABLE 1:** Statistics of draft genome assembly of *P. granatum*.

Statistics without reference	*P. granatum* references (GCF_007655135)	*P. granatum* draft assembly
Estimated genome size (Mb)	313.18 Mb	352.38 Mb
Assembled genome size (Mb)	320.31 Mb	361.76 Mb
Number of scaffolds (≥1 kb)	473	122
N50 scaffold length (Mb)	39.02	39.79
Longest scaffold (Mb)	54.25	86.06
Total size of assembled contigs (Mb)	320.33 Mb	361.76 Mb
Number of contigs (≥1 kb)	661	7,640
Number of contigs (≥ 50,000 bp)	399	1,555
N50 contig length (kb)	4,489.929	61.031
Largest contig (kb)	14,772.832 kb	487.599 kb
Total length	320494280	361760465
N50 length (Mb)	39.95 Mb	40.75 Mb
GC (%)	40.38	38.86
Number of genes	33,594	30,803

### BUSCO Analysis

The quantitative assessment of *P. granatum* draft *de novo* hybrid genome assembly and annotation completeness carried out using BUSCO is represented in Table 2. The draft assembly consists of 90.6% of complete BUSCOs and a very minute percentage of missing, fragmented, and duplicated BUSCOs, indicating the completeness of assembled genome. Further, the draft genome completeness is comparable to that of all other assemblies of *P. granatum* available at NCBI.

**TABLE 2:** Qualitative analysis of draft genome assembly.

Measures	*P. granatum* draft assembly	*P. granatum* cultivar Dabenzi	*P. granatum* cultivar Taishanhong	*P. granatum* isolate Tunisia 2019	*P. granatum* strain AG2017
No. (percentage) of complete BUSCOs (C)	2,114 (90.9%)	2,156 (92.7%)	2,159 (92.8%)	2,150 (92.5%)	2,114 (90.9%)
No. (percentage) of complete and single-copy BUSCOs (S)	2,012 (86.5%)	2,096 (90.1%)	2,099 (90.2%)	2,069 (89.0%)	2,062 (88.7%)
No. (percentage) of complete and duplicated BUSCOs (D)	102 (4.4%)	60 (2.6%)	60 (2.6%)	81 (3.5%)	52 (2.2%)
No. (percentage) of fragmented BUSCOs (F)	91 (3.9%)	79 (3.4%)	76 (3.3%)	84 (3.6%)	93 (4.0%)
No. (percentage) of missing BUSCOs (M)	121 (5.2%)	91 (3.9%)	91 (3.9%)	92 (3.9%)	119 (5.1%)
Total BUSCO groups searched	2,326	2,326	2,326	2,326	2,326

BUSCO (Benchmarking set of universal single-copy orthologues) result for draft assembly, Dabenzi, Taishanhong, isolate Tunisia 2019, and strain AG2017 of P. granatum.

#### Transposable Elements in the Genome

The whole-genome data were also used to identify the transpositional landscape of pomegranate, represented in Table 3. *P. granatum* genome harbors both Type I and Type II transposable element (TE) families. The repeat elements occupy only 26.68% of the genome. Long terminal repeat (LTR) retroelements are the most abundant TEs, representing 11.43% of the assembly. Among the LTR elements, Copia/TY1 are more copious than Gypsy/DIRS1.

**TABLE 3:** Types of transposable elements identified in *P. granatum* genome. The total interspresed repeats mentioned at the bottom of the table 26.68 is the total of retroelements (13.69), DNA transposons (11.23) and unclassified (1.76) Hence these values are highlighted.

Types of Transposable element	Number of elements	Length occupied in bp	Percentage of sequences
Retroelements	112,404	49,529,468	**13.69**
SINEs	13,048	1,498,787	0.41
LINEs	25,893	6,693,226	1.85
(i) CRE/SLACS	0	0	0
(ii) L2/CR1/Rex	0	0	0
(iii)R1/LOA/Jockey	21	5,707	0
(iv) R2/R4/NeSL	0	0	0
(v)RTE/Bov-B	0	0	0
(vi)L1/CIN4	3,843	1,720,126	0.48
LTR elements	73,463	41,337,455	11.43
(i) BEL/Pao	0	0	0
(ii) Ty1/Copia	8,660	5,573,403	1.54
(iii) Gypsy/DIRS1	3,262	2,504,146	0.69
(iv) Retroviral	0	0	0
DNA transposons	182,314	40,633,158	**11.23**
(i) hobo-Activator	683	418,562	0.12
(ii) Tc1-IS630-Pogo	24	11,586	0
(iii) En-Spm	0	0	0
(iv) MuDR-IS905	0	0	0
(v) PiggyBac	0	0	0
(vi) Tourist/Harbinger	954	607,183	0.17
(vii) Other (Mirage,P-element, Transib)	0	0	
Rolling-circles	835	750,813	0.21
(i)Unclassified	30,347	6,356,886	**1.76**
Total interspersed repeats		96,519,512	**26.68**

#### Microsatellites Predicted in the Genome

**FIGURE 2:**
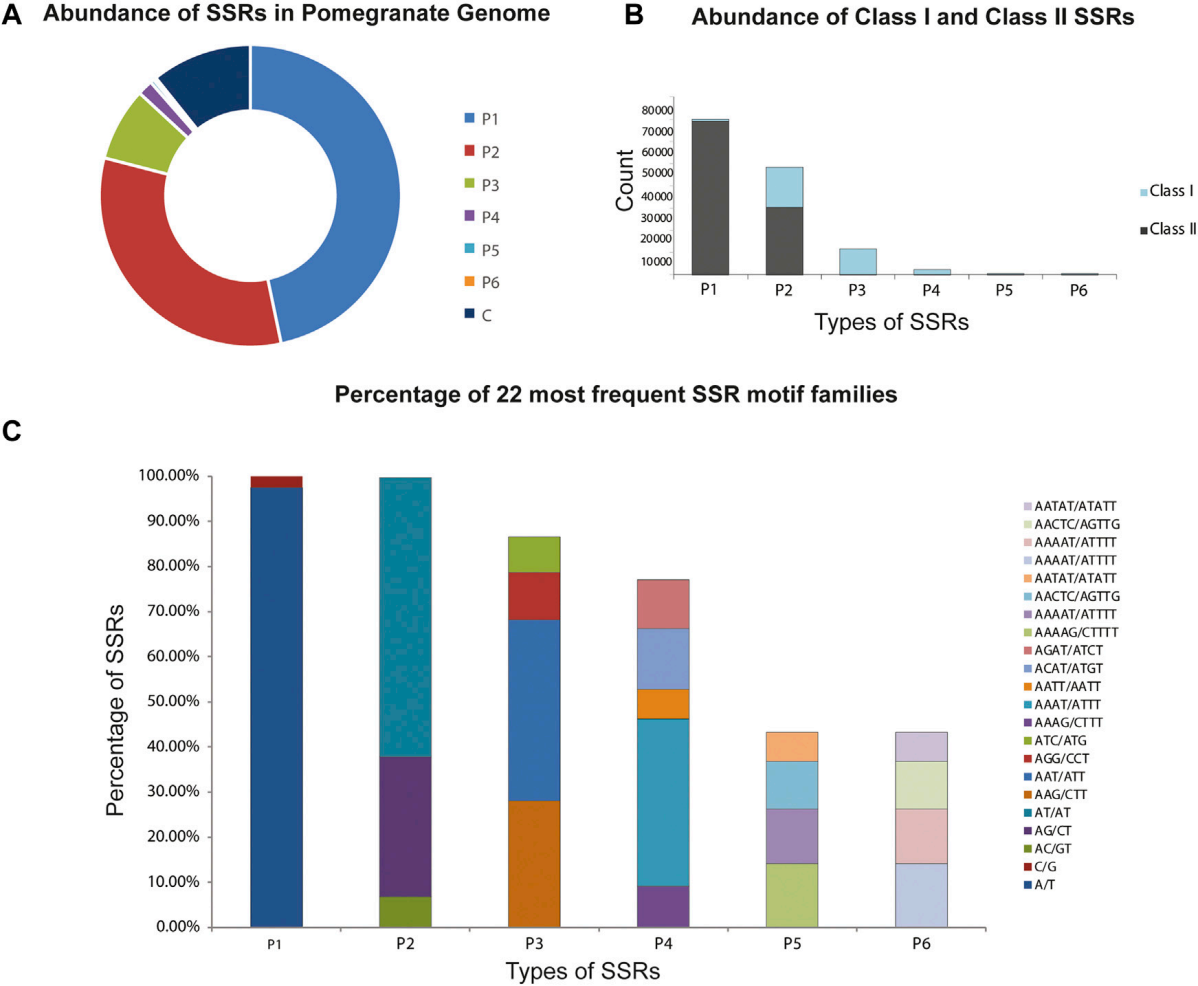
Classification and distribution of microsatellites alias SSRs identified in the *P. granatum* genome. **(A)** Proportions of microsatellites with different motif types. P1: mono-nucleotide repeats; P2: di-nucleotide repeats; P3: tri-nucleotide repeats, P4: tetra-nucleotide repeats; P5: penta-nucleotide repeats; p6: hexa-nucleotide repeats; C: complex: no. of SSRs involved in compound formation. **(B)** Percentage of hypervariable class I and variable class II microsatellites in the *P. granatum* genome. **(C)** Frequency of distribution of the most frequently occurring SSR motif families.

A total of 133827 SSRs were revealed in the *P. granatum* genome with a relative abundance of 369.93 SSRs/Mb. The total length of the complete set of microsatellites was 0.06% of the assembled genome. The most frequent motifs were mono- (52.4%) followed by di- (36.2%), tri- (8.8%), tetra- (1.8%), penta- (0.5%), and hexa-nucleotides (0.3%). The genome sequence also yielded both complex and compound SSRs with an overall density of 0.12%. It was also observed that SSRs composed of smaller repeats accounted for a larger percentage, while those with large repeats represented a smaller percentage of SSRs (Figure 2A). The P1, P2, P3, P4, P5, and P6 were divided into two classes based on the SSR length. A total of 25.6% and 75.3% were classified into long and hypervariable class I type SSRs (≥20 bp) and class II type (10–19 bp), respectively (Figure 2B). The distribution of the different SSR types is heterogeneous, particularly in mono- and di-nucleotides. Among P1, T/A (97.6%) were most frequently occurring and were also the most frequent motif in the entire genome, accounting for 51.0%, and G/C (2.4%) were in almost negligible amounts. Among P2, the highly distributed motifs were AT/AT (61.9%) and AG/CT (31.0%). AAT/ATT (40.1%) and AAG/CTT (28.0%) motifs were the most abundant. Among P4, AAAT/ATTT (37.1%), P5 AAAAG/CTTTT (14.2%), AAAAAT/ATTTTT (12.1%), and P6 AAAATC/ATTTTG (34.5%) and AAAAAG/CTTTTT (10.2%) were the most abundant repeats in P3, P4, P5, and P6 classes (Figure 2C).

#### Gene Predictions and Annotations

**FIGURE 3:**
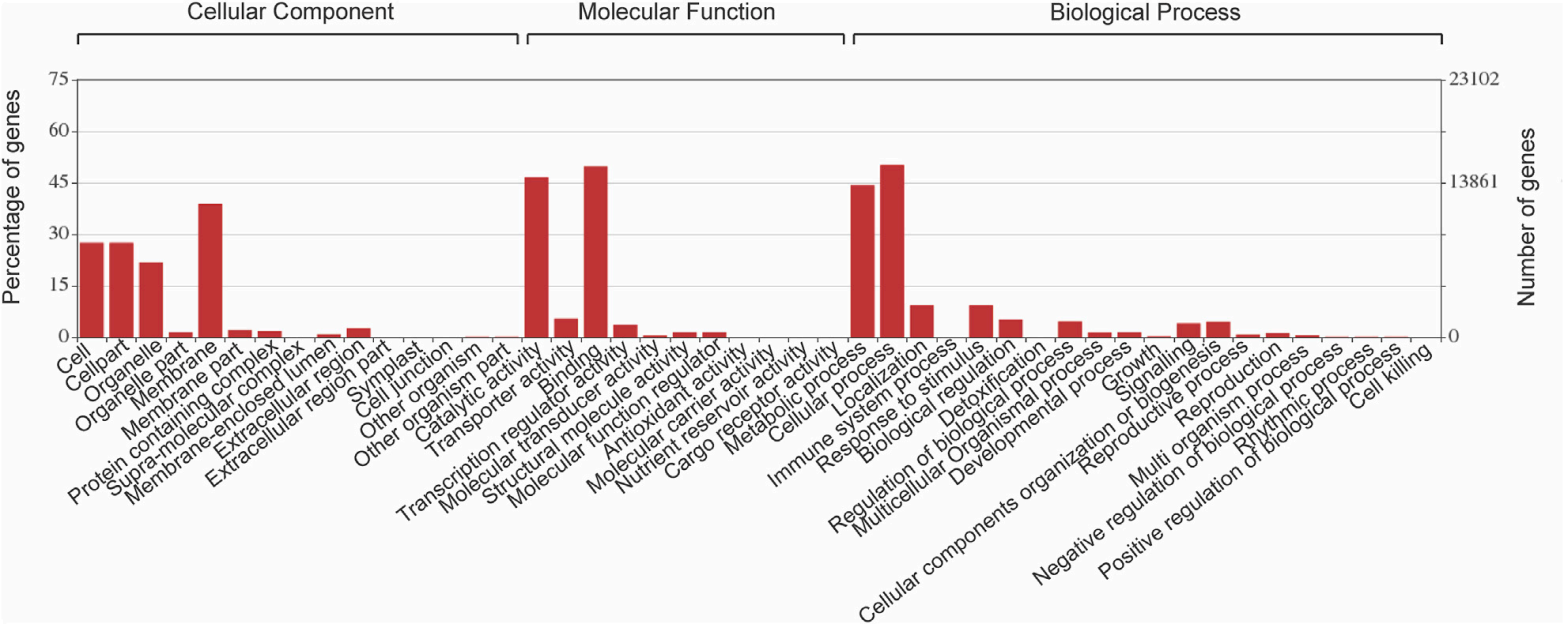
Bar chart exhibits gene annotations of the functional classes in each of the three major categories, biological process (BP), cellular component (CC), and molecular function (MF), of gene ontology classification.

Comprehensive gene predictions and putative functions assignment of *P. granatum* were made by comparing against the eudicotyledons of NCBI NR (non-redundant) database. The obtained hits were further annotated using BLAST2GO, and 30,803 genes were identified. These 30,803 protein sequences were classified into 30 functional classes under three core categories: biological processes (BP), cellular components (CC), and molecular functions (MF), based on homology (Figure 3) (Supplementary Table S2). On closer examination of the species distribution of hits in the UniportKB database, maximum hits were from the *Eucalyptus grandis* plant*.*

### Elucidation of Metabolic Pathways

KEGG Orthology (KO) links directly to known pathways and KO annotations facilitate concurrent pathway identification. Therefore, 30,803 annotated proteins were mapped to 128 reference pathways. All proteins were classified first at the top three levels: cellular component (18,152), molecular function (23,522), and biological process (19,029). The three functional categories are divided into 46 sub-categories corresponding to the KEGG pathways. 274 and 171 KEGG annotated proteins mapped under the category of biosynthesis of secondary metabolites and terpenoids, respectively (Supplementary Table S3). Because various pharmacological properties of pomegranate are attributed to the presence of secondary metabolites produced in different parts of the plant, we sorted 4,446 proteins coding genes for phenylpropanoid and flavonoid biosynthesis pathways.

#### Phenylpropanoid Biosynthesis Pathway

**FIGURE 4:**
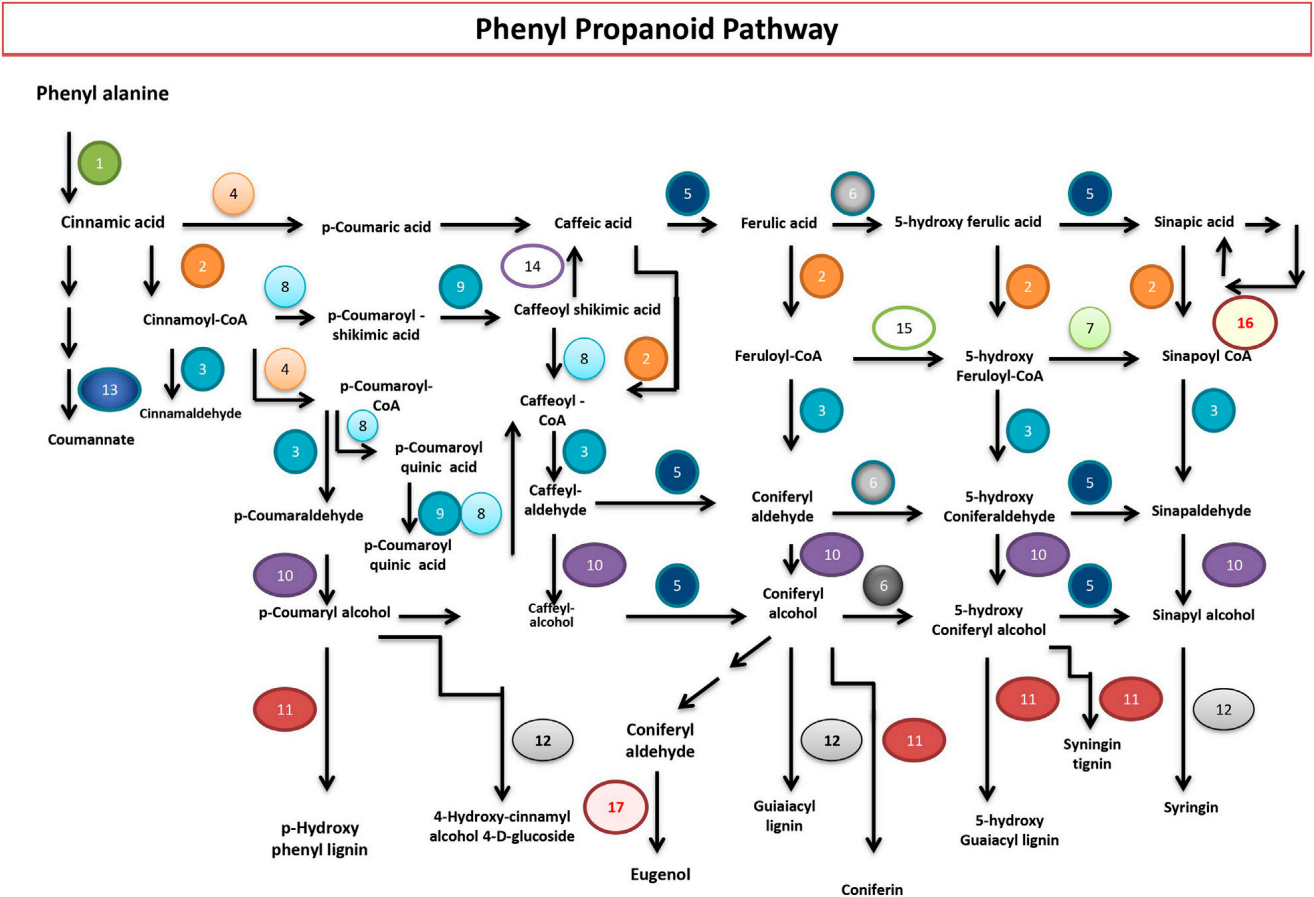
Phenylpropanoid biosynthesis pathway in *P. granatum*. Numbers 1 to 17 represent the enzymes that catalyze the respective reactions. 1) PAL; phenylalanine ammonia-lyase [EC:4.3.1.24]; 2) 4CL; 4-coumarate--CoA ligase [EC:6.2.1.12]; 3) CCR; cinnamoyl-CoA reductase [EC:1.2.1.44]; 4) CYP73A; trans-cinnamate 4-monooxygenase [EC:1.14.13.11]; 5) E2.1.1.68; caffeic acid 3-O-methyltransferase [EC:2.1.1.68]; 6) CYP84A; ferulate-5-hydroxylase [EC:1.14.-.-]; 7) E2.1.1.104; caffeoyl-CoA O-methyltransferase [EC:2.1.1.104]; 8) E2.3.1.133; shikimate O-hydroxycinnamoyltransferase [EC:2.3.1.133]; 9) CYP98A; coumaroylquinate (coumaroylshikimate) 3′-monooxygenase [EC:1.14.13.36]; 10) E1.1.1.195; cinnamyl-alcohol dehydrogenase [EC:1.1.1.195]; 11) E1.11.1.7; peroxidase [EC:1.11.1.7]; 12) UGT72E; coniferyl-alcohol glucosyltransferase [EC:2.4.1.111]; 13) bglB; beta-glucosidase [EC:3.2.1.21]; 14) CSE; caffeoylshikimate esterase [EC:3.1.1.-]; 15) REF1; coniferyl-aldehyde dehydrogenase [EC:1.2.1.68]; 16) serine carboxypeptidase-like 19 [EC:3.4.16.- 2.3.1.91]; 17) eugenol synthase [EC:1.1.1.318].

The phenylpropanoid biosynthesis pathway (PP) is essential for a plant’s growth, development, and defense. It saddles between the primary and the secondary metabolism. The PP starts with phenylalanine, the end product of the shikimate pathway. Phenylalanine ammonia-lyase (PAL) transforms phenylalanine into cinnamic acid, which leads to the formation of p-coumaric acid by the enzymatic action of trans-cinnamate 4-monooxygenase, which then transforms into p-coumaroyl-CoA by p-coumaroyl: CoA ligase (4CL). Both p-coumaric acid and p-coumaroyl-CoA can act as precursors of the lignin monomer pathway. In contrast, p-coumaroyl-CoA is the precursor of the flavonoid, stilbenoid, diarylheptanoid, and gingerol biosynthetic pathways. The current study identified genes that direct the biosynthesis of monolignols and hydroxycinnamic acids, such as ferulic and sinapic acids, and their corresponding esters. Lignin confers pathogen resistance, vascular integrity, and structural support. Genes coding for enzymes involved in the synthesis of all the three monolignols (p-hydroxycinnamyl alcohols): p-coumaryl, coniferyl, and sinapyl alcohols that result in the lignin polymer were identified in abundance. The 17 enzymes identified are PAL; phenylalanine ammonia-lyase [EC:4.3.1.24], 4CL; 4-coumarate--CoA ligase [EC:6.2.1.12], CCR; cinnamoyl-CoA reductase [EC:1.2.1.44], CYP73A; trans-cinnamate 4-monooxygenase [EC:1.14.13.11], caffeic acid 3-O-methyltransferase [EC:2.1.1.68], CYP84A; ferulate-5-hydroxylase [EC:1.14.-.-], caffeoyl-CoA O-methyltransferase [EC:2.1.1.104], shikimate O-hydroxycinnamoyltransferase [EC:2.3.1.133], coumaroylquinate (coumaroylshikimate) 3′-monooxygenase [EC:1.14.13.36], cinnamyl-alcohol dehydrogenase [EC:1.1.1.195], peroxidase [EC:1.11.1.7], coniferyl-alcohol glucosyltransferase [EC:2.4.1.111], beta-glucosidase [EC:3.2.1.21], caffeoyl shikimate esterase [EC:3.1.1.-], and coniferyl-aldehyde dehydrogenase [EC:1.2.1.68], serine carboxypeptidase-like 19 [EC:3.4.16.- 2.3.1.91], and eugenol synthase [EC:1.1.1.318] (Figure 4).

#### Flavonoid Biosynthesis Pathway

**FIGURE 5:**
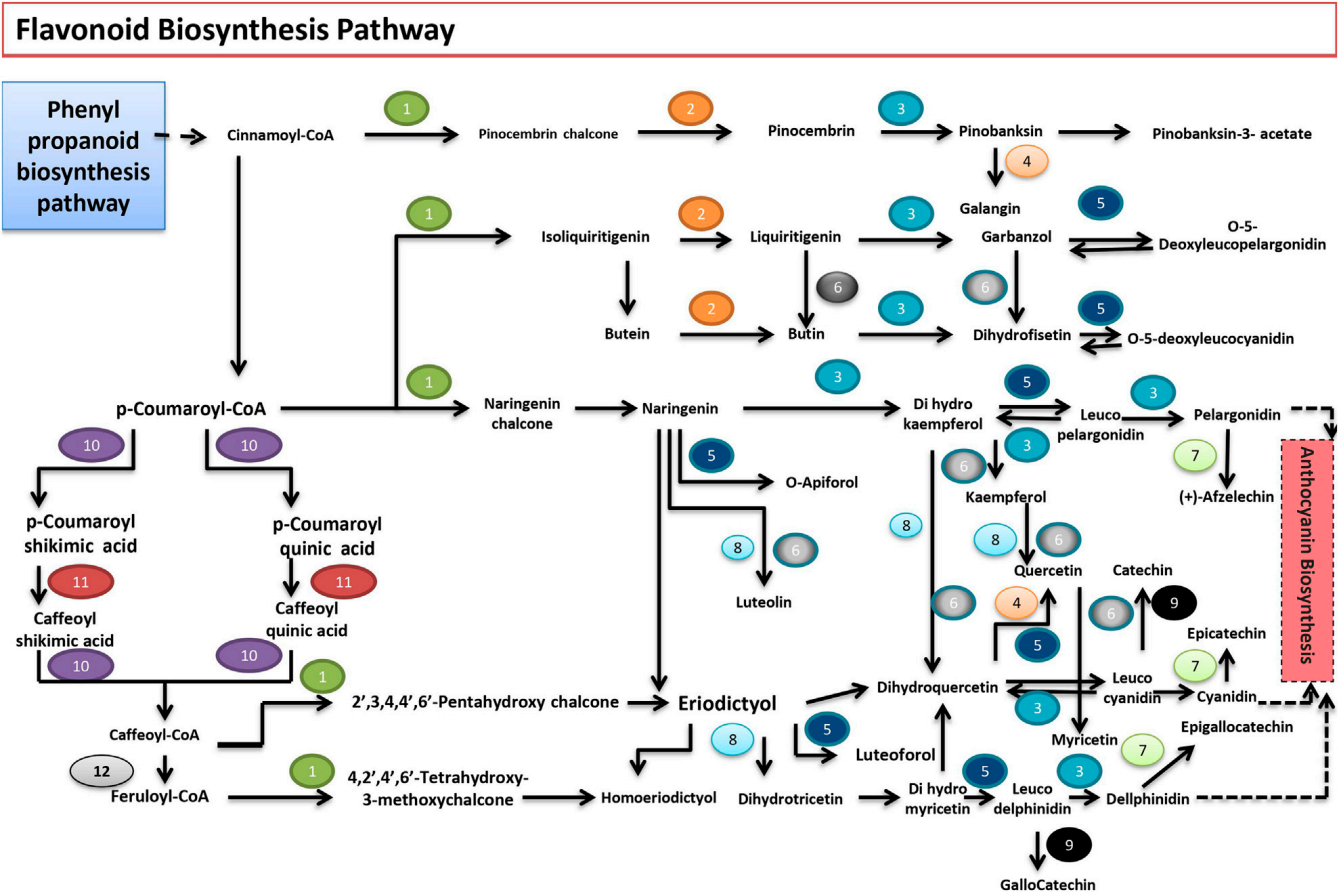
Flavonoid biosynthetic pathway found in *P. granatum.* Numbers 1 to 14 represent the enzymes that catalyze the respective reactions. 1) CHS; chalcone synthase [EC:2.3.1.74]; 2) E5.5.1.6; chalcone isomerase [EC:5.5.1.6]; 3) E1.14.11.9; naringenin 3-dioxygenase [EC:1.14.11.9]; 4) FLS; flavonol synthase [EC:1.14.11.23]; 5) DFR; bifunctional dihydroflavonol 4-reductase/flavanone 4-reductase [EC:1.1.1.219 1.1.1.234]; 6) E1.14.13.21; flavonoid 3′-monooxygenase [EC:1.14.13.21]; 7) ANR; anthocyanidin reductase [EC:1.3.1.77]; 8) CYP75A; flavonoid 3′,5′-hydroxylase [EC:1.14.13.88]; 9) LAR; leucoanthocyanidin reductase [EC:1.17.1.3]; 10) E2.3.1.133; shikimate O-hydroxycinnamoyltransferase [EC:2.3.1.133]; 11) CYP98A; coumaroylquinate (coumaroylshikimate) 3′-monooxygenase [EC:1.14.13.36]; 12) E2.1.1.104; caffeoyl-CoA O-methyltransferase [EC:2.1.1.104]; 13) CYP73A; trans-cinnamate 4-monooxygenase [EC:1.14.13.11]; 14) E1.14.11.19; leucoanthocyanidin dioxygenase [EC:1.14.11.19].

The flavonoids can be classified into six major groups. These compounds impart protection against exposure to ultraviolet (UV) radiation and phytopathogens, help in signaling during nodulation, male fertility, auxin transport, and coloration of flowers, a visual indicator for pollinators. The draft genome of *P. granatum* revealed the presence of 14 genes capable of encoding enzymes for the flavonoid pathway. Like many other plant species, flavonoids are synthesized through the phenylpropanoid pathway in *P. granatum*. Cinnamoyl CoA or p-coumaroyl-CoA serves as a precursor of the flavonoid biosynthesis pathway. The first enzyme specific for the flavonoid pathway, chalcone synthase, produces naringenin chalcone and the formation of stereospecific cyclic naringenin catalyzed by chalcone isomerase. Naringenin is converted to dihydrokaempferol by naringenin 3-dioxygenase. The bifunctional dihydroflavonol-4-reductase catalyzes the reduction of dihydrokaempferol to leucopelargonidin, which is further converted to pelargonidin, an anthocyanidin, by the action of anthocyanidin synthase. The anthocyanidins are reduced to flavan 3-ols (e.g., catechin and epicatechin) by leucoanthocyanidin reductase (LAR) and anthocyanidin reductase (ANR), respectively. The dihydroflavonol (i.e., the dihydrokaempferol) is converted into kaempferol by flavonol synthase. Flavonoid 3′,5′-hydroxylase catalyzes the formation of quercetin from kaempferol. Flavonoid 3′,5′-hydroxylase enzyme acts on naringenin, eriodictyol, dihydroquercetin, and dihydrokaempferol. Flavone synthase I (FNS I) or flavone synthase II (FNS II) synthesizes luteolin from naringenin. P-coumaroyl-CoA is converted to feruloyl coA with the help of coumaroylquinate (coumaroylshikimate) 3′-monooxygenase and caffeoyl-CoA O-methyltransferase (Figure 5). The presence of genes coding for these proteins in the genome sequence was also confirmed in this pathway analysis.

### Comparative Genome Analysis of *P. granatum* and Other Eudicot Species

**FIGURE 6:**
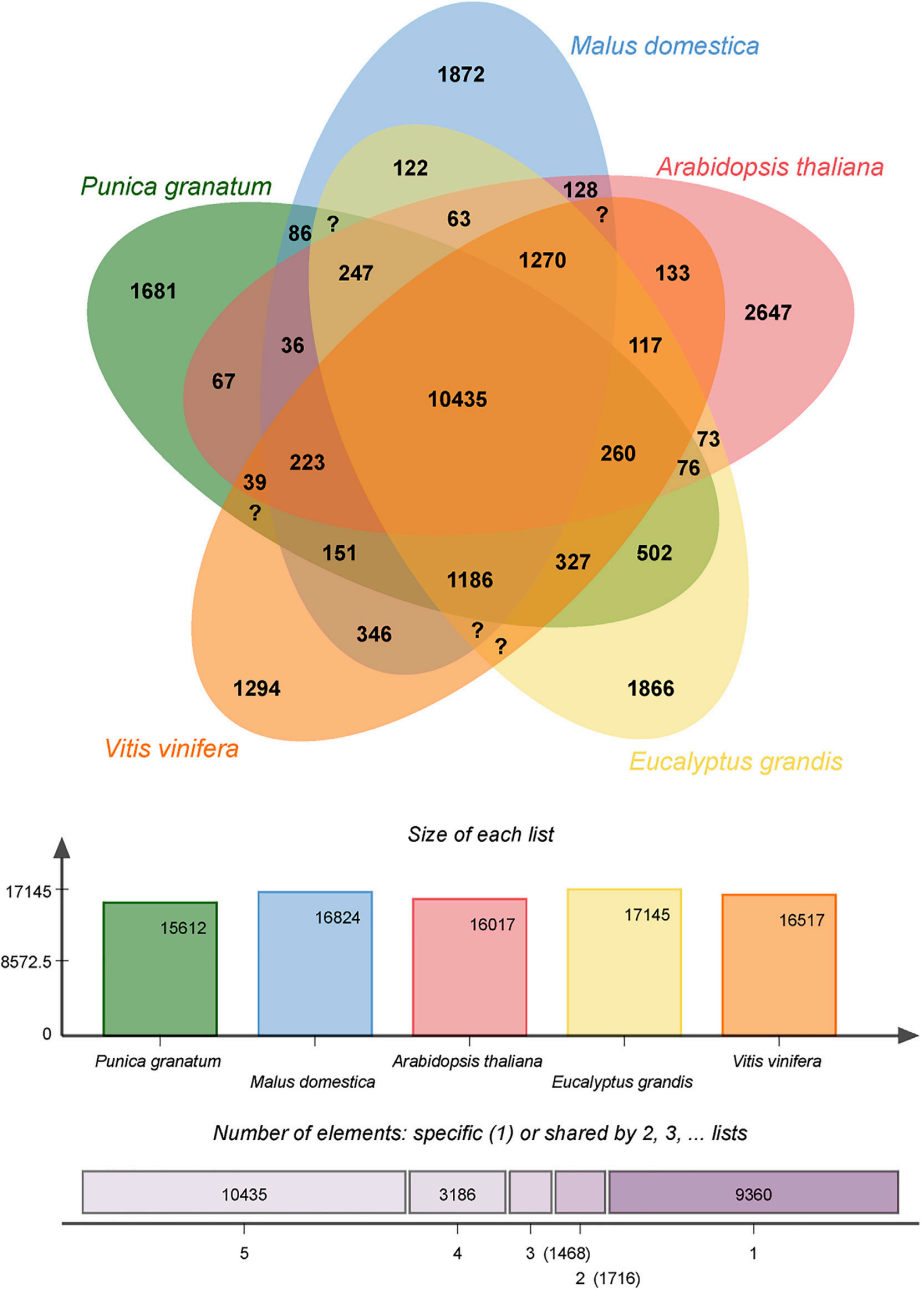
Venn diagram of shared orthologous gene families in *Punica granatum*, *Eucalyptus grandis*, *Malus domestica*, *Vitis vinifera*, and *Arabidopsis thaliana*. The gene family number is listed in each component.

The full gene sets of pomegranate (*Punica granatum*), apple (*Malus domestica*), *Arabidopsis* (*Arabidopsis thaliana*), grape (*Vitis vinifera*), and *Eucalyptus* (*Eucalyptus grandis*) were analyzed using a gene family cluster analysis and is depicted in Figure 6. The pomegranate genome has 30,803 genes organized into 15,612 gene clusters, 10,435 of which are shared by all five species, advocating their conservation in the lineage after speciation. *P. granatum* shared more gene family clusters (15,316) with *E. grandis* than any of the other three species. Moreover, 1,681 clusters were unique to *P. granatum*. The gene clusters within many genes or in-paralog clusters are most likely the sources of these clusters. The presence of in-paralog clusters suggests that certain gene families in *P. granatum* may have undergone lineage-specific gene expansion. According to the annotation of these clusters, some of these lineage-specific clusters may be involved in key biological processes such as cellular processes, genetic information processing, metabolism, biosynthesis of secondary metabolites, and environmental information processing. The biosynthesis of indole alkaloid, phenylpropanoid, flavonoid, anthocyanin, isoflavonoid, stilbenoid, gingerol, isoquinoline alkaloids, tropane, piperidine and pyridine alkaloid, and glucosinolate genes are identified. However, the present study focused only on phenylpropanoid, flavonoid biosynthesis due to the therapeutic benefits of these secondary metabolites.

### Phylogenetic Tree Based on Average Nucleotide Identity Analysis

**FIGURE 7:**
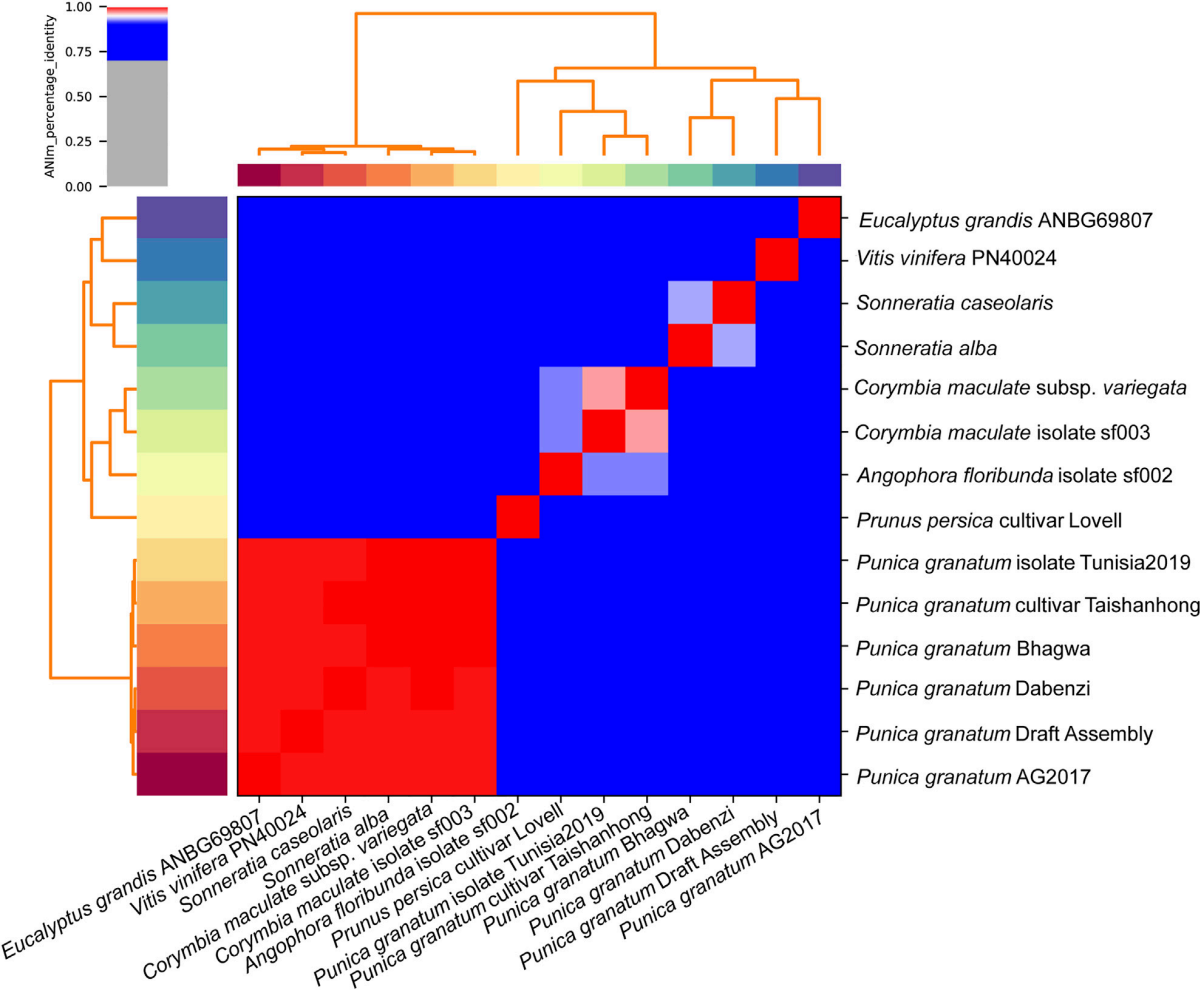
Heatmap of ANIm percentage identity: species-level assignments and isolate identifiers as indicated at source given as row and column labels. Cells in the heatmap corresponding to 95% ANIm sequence identity are colored red. Blue cells correspond to ANIm comparisons indicating that the corresponding organisms do not belong to the same species. Color intensity fades as the comparisons approach 95% ANIm sequence identity. Color bars above and to the left of the heatmap correspond to source species-level assignments for each isolate in the analysis. Hierarchical clustering of the analysis results in two dimensions is represented by dendrograms, constructed by simple linkage of ANIm percentage identities.

A hybrid draft genome of *P. granatum* was built using MaSURCA and WENGAN. We compared it to genome sequences of *P. persica* (cultivar_Lovell), *P. granatum* (cultivar Bhagwa, Dabenzi, Taishanhong, Tunisia 2019, and strain AG 2017), *S. alba*, *V. vinifera* (cultivar PN40024), *S. caseolaris*, *E. grandis* (isolate ANBG69807), *C. maculata* (isolate sf003), *A. floribunda* (isolate sf002), and *C. citriodora* (subsp. variegata) The *P. granatum* cultivar Bhagwa was closely related to other *Punica* varieties such as Taishanhong and Tunisia 2019 (ANIm >99%). *P. granatum* cultivar Dabenzi, draft assembly, and strain AG2017 also share the same clad and exhibit 99% genetic identity according to average nucleotide analysis using ANI tool PyANI (ANIm) (Figure 7). ANIm genome comparison confirmed that *Eucalyptus grandis* and *Vitis vinifera* are distant relatives of *P. granatum* cultivar Bhagwa falling into separate clad, splitting from other clade members.

### Identification and Analysis of Variants in *P. granatum G*enome

Variant analysis using the representative genome of *P. granatum* (accession number: GCF_007655135.1_ASM765513v2) from NCBI revealed that the variants are categorized as 359,607 (80.35%) SNPs, 39,857 (8.90%) deletions, and 48,074 (10.74%) insertions. Considering the variants categorized based on the impact, 1,008,520 (95.146%) were classified as modifiers, 28,258 (2.666%) as moderate, and 20,275 (1.913%) as low impact variants. While distributions of the variants based on functional class revealed 27,287 (61.696%) as missense mutations, 16,585 (37.499%) as silent, and 356 (0.805%) as nonsense mutations, the counts based on the effects showed 358,049 (33.668%) in the intergenic region, 100,729 (9.472%) as intron variants, 27,146 (2.553%) missense variants, 16,571 (1.558%) synonymous variants, and 13,546 (1.274%) 3′-UTR variants. The variants were classified based on their putative effect on annotated genes, and gene ontology analysis was carried out to acquire a deeper understanding of the functions of 10 genes affected by large-effect variants. These 10 protein sequences were classified into 18 functional classes under three core categories: biological processes (BP), cellular components (CC), and molecular functions (MF), based on homology (Supplementary Table S4).

## Discussion

*P. granatum*, the crown jewel of the fruit world, is highly nutritious, and produces a phenomenal amount of phytopharmaceuticals and nutraceuticals. The whole draft genome described here in this study using a hybrid approach will serve as a helpful resource in identifying genes and determining their functions. In any genome sequencing project, the foremost requirement is genome size estimation, as it aids in planning the genomic library construction and deciding the amount of raw sequence data to be collected. The nuclear genome size of a plant species is also one of the seminal characteristics, which provides a basic understanding of its cytogenetic features, taxonomic location, and evolution. It is also used to validate the completeness of whole-genome assemblies (Al-Qurainy et al., 2021). However, primary cytogenetic data is unavailable for *P. granatum* “Bhagwa.” Therefore, first, we estimated the genome size of *P. granatum* “Bhagwa” using the flow cytometry method, which was determined to be ∼352.38 Mb, ∼76 Mb larger than the estimated size of 276.6 Mb predicted by GenomeScope, based on Illumina paired-end data.

The whole-genome *de novo* assembly is a critical step in genome research. It is decisive in drawing further genetic resources such as gene annotations, repeat element predictions, and pathway predictions. The datasets obtained using two next-generation sequencing (NGS) techniques and subsequent assembly using several assemblers using various algorithms revealed a difference in genome size estimation using K-mer and flow cytometric analysis. This invoked the need for determining a more reliable and capable assembler for the obtained pomegranate plant genomic data using comparative sequence analysis. The assembled genome size of the *P. granatum* “Bhagwa” (361.76 Mb) is slightly larger than that of the Chinese varieties of pomegranate “Dabenzi” (328.38 Mb) (Qin et al., 2017), “Taishanhong” (274 Mb) (Yuan et al., 2018), and “Tunisia” (320.31 Mb) (Luo et al., 2020). The draft genome completeness is comparable to all other assemblies of *P. granatum* available at NCBI.

In a genome project, genome assembly is succeeded by an ensemble of gene annotations to gain insights into the plant’s taxonomy, development, evolution, and functional and metabolic potential (Josep et al., 2019). TEs exhibit high diversity in structure and modes of transposition and play a vital role in genome evolution, gene regulation, and epigenetics (Sahebi et al., 2018). The TEs in the *P. granatum* genome occupy only a minority (26.68%) of the genome. This landscape is low compared to other pomegranate cultivars, notably with a burst of TE amplification evident in China cultivars (Supplementary Table S5). This minor quantity reported could be due to the use of hybrid sequencing technology, including long-read technology in the present study. The long-read technology can overcome the low resolution of reconstructing repetitive regions. In general, the quantity of LTR-REs mostly correlates with the genome size of the plant species (Zedek et al., 2010). The small genome size of the angiosperm *P. granatum* “Bhagwa” correlates well with the proportion of TEs. Retroelements are the predominant elements other than DNA transposons (Bourque et al., 2018). In *P. granatum*, the *Copia* is more abundant than *Gypsy*, with the LTR Res elements present in large numbers. Much of the adaptation and growth of *P. granatum* in various agro-climatic conditions can be attributed to TEs domesticated by the pomegranate genome (Makarevitch et al., 2015). On the other side, the TEs could also contribute to variation in the genome size of various cultivars (SanMiguel et al., 1998). Our results reveal lesser TEs than in other cultivars such as Thaishanhong (Yuan et al., 2018) and Dabenzi (Luo et al., 2020). This observation indicates that different genomes of various cultivars of pomegranate show unique TEs expansion patterns due to other evolutionary processes.

The characterization of the SSRs in *P. granatum* revealed that P1 repeats were the most predominant repeats. SSR frequency decreased with an increase in repeat units in the *P. granatum* genome, which is comparable to that documented in monocots (*Brachypodium*, *Sorghum*, and rice) and dicots (*Arabidopsis*, *Medicago*, and *Populus*) (Sonah et al., 2011). Genome-wide analysis of SSRs is expected to provide insights into quantitative trait loci (QTL) based selection, plant breeding, genetic linkage mapping, population, and evolutionary genetics of *P. granatum.* The relative abundance of SSRs is relatively high with 369.93 SSRs/Mb and in trend with microsatellite frequency, which is higher in small genomes and is lower in large genomes. This higher density can be expected because of the mutational effects of replication slippage (Morgante et al., 2002).

The deluge of data from modern genomics technologies empowered research on the biosynthesis and regulation of diverse plant secondary metabolites (Kim and Buell, 2015). Gene annotations resulted in the prediction of a total of 30,803 proteins. Examination of the genome sequence of *P. granatum* also enabled the identification of genomic signatures of secondary metabolism genes of phenylpropanoid (17 genes) and flavonoid (14 genes) pathways. *P. granatum* is a good source of p-coumaric acid and is one of the very important phytopharmaceuticals with anti-breast cancer activity, as reported in one of the earlier studies (Usha et al., 2021). Notably, it is also a precursor molecule for flavonoid biosynthesis (Ferreyra et al., 2012). The *P. granatum* produces a high amount of polyphenols and flavonoids (Middha et al., 2013a), which highly correlates with the existence of a more significant amount of phenylpropanoid and flavonoid pathway genes in *P. granatum* “Bhagwa.” The enzymes that catalyze the synthesis of major flavonoids, cyanidin, epicatechin, kaempferol, luteolin, naringin, pelargonidin, and quercetin, were identified. These flavonoids exhibit antioxidant, antineoplastic, anti-inflammatory, antiviral, and antibacterial activities (Middha et al., 2013b). The genome sequence of *P. granatum* “Dabenzi” provides information on candidate genes for punicalagin biosynthesis, which mainly catalyzes gallic acid synthesis from shikimic acid and syringate, not from quinic acid (Qin et al., 2017). These results coincide with our findings that phenylpropanoids are synthesized from shikimic acid in *P. granatum* “Bhagwa” species. Consistent with prior reports, the recent common ancestry between *E*. *grandis* and *P*. *granatum* was observed through comparative genome analysis studies of clustered single-copy gene orthologs related to secondary metabolism (Qin et al., 2017). The current study illustrates how hybrid sequencing technology can resolve complex TE and SSRs. These regions encompass essential genomic information critical for the adaptation and evolution to the environment, thus assisting in developing the crops by genetic breeding methodologies. The draft genome and its annotations of *P. granatum* “Bhagwa” will accelerate crop improvement by selecting desirable genes with enhanced agronomic traits, including nutrient richness, high yield, biotic and abiotic stress tolerance, and resistance against pathogens, and high yield of secondary metabolites with pharmacological properties.

## Conclusion

The assembled first draft genome of *P. granatum* “Bhagwa” in this study can be a valuable resource and reference to understanding this commercially important fruit crop’s taxonomy, evolution, and biological architecture. The study also provides a novel experimental (hybrids sequencing technology) and computational approach of using multiple assemblers for dealing with the difference between the flow cytometric and k-mer genome size estimations. The vast data and information obtained from the draft genome of *P. granatum* will also improve the crop identification and understanding of the biosynthesis of phytopharmaceuticals. Unraveling the high-quality complete genome and transcriptome of the *P. granatum* will further facilitate future research into other areas of research on this plant, such as aspects of its environmental stress tolerance, acclimatization, evolution, biosynthesis of other pharmacologically important secondary metabolites, crop improvement, and resistance to pathogens.

## Data Availability

All the raw data used in this study was submitted to the NCBI SRA data repository (BioSample: SAMN07645014; Sample name: Pomegranate (*Punica granatum*); SRA: SRS2645763) (https://www.ncbi.nlm.nih.gov/bioproject/PRJNA407279) under the accession number. All secondary data used in this study are available in the Supplementary Materials provided.

## Footnotes

1
https://clinicaltrials.gov/ct2/results?term=pomegranateandSearch=Applyandrecrs=eandage_v=andgndr=andtype=andrslt=.

2
https://github.com/rrwick/Filtlong)(parameters --min_length 500 --keep_percent 90.

3
http://www.genome.umd.edu/masurca.html.

4
http://www.repeatmasker.org/RMBlast.html.

6
https://github.com/mnenno/PySSRstat.

7
https://github.com/tseemann/barrnap.

8
http://ccb.jhu.edu/software/stringtie/gff.shtml#gffread.

9
https://www.genome.jp/kegg/kaas/.

10
https://gatk.broadinstitute.org/hc/en-us.
