# Supplementary material for: Hybrid Assembly and Annotation of the Genome of the Indian Punica granatum, a Superfood
Source: Front Genet. 2022 May 11;13:786825. doi: 10.3389/fgene.2022.786825 (PMC9130716; doi:10.3389/fgene.2022.786825)
Supplement: Supplementary file 2 [file Table1.docx]

| **Library name and Type** | **Raw Read** | **Read Length** | **Total Raw Data (Nucleotide size)** | **Trimmed Read** | **%GC** | **Coverage**  **(X)** |
| --- | --- | --- | --- | --- | --- | --- |
| ***Data obtained through Illumina sequencing technology*** | | | | | | |
| Pomegranate_R1.fastq (Paired end) | 174115936 | 150*2 | 26117390400 | 135042241 | 42 | 72.33 |
| Pomegranate_R2.fastq (Paired end) | 174115936 | 150*2 | 26117390400 | 135042241 | 42 | 72.33 |
| Pomegranate_MP_3-5Kb_R1.fastq  (Mate-pair) | 37847829 | 150*2 | 5715022179 | 12045895 | 46 | 3.13 |
| Pomegranate_MP_3-5Kb_R2.fastq  (Mate-pair) | 37847829 | 150*2 | 5715022179 | 12045895 | 46 | 2.97 |
|  |  |  | **63589129500** |  |  | **150.76** |
|  |  |  | **63.5 Gb** |  |  |  |
|  | | | | | | |
| ***Data obtained through Oxford Nanopore sequencing technology*** | | | | | | |
| Nanopore_Reads.fasta | 554163 | 2319.56 |  |  | 46 | 4.32 |
|  |  |  |  |  |  | 155.08 |

**Supplementary Table S1.** Statistics of raw and processed data obtained from Illumina and Oxford Nanopore sequencing technologies.
