# Supplementary material for: Hybrid Assembly and Annotation of the Genome of the Indian Punica granatum, a Superfood
Source: Front Genet. 2022 May 11;13:786825. doi: 10.3389/fgene.2022.786825 (PMC9130716; doi:10.3389/fgene.2022.786825)
Supplement: Supplementary file 3 [file DataSheet1.doc]

Supplementary Material


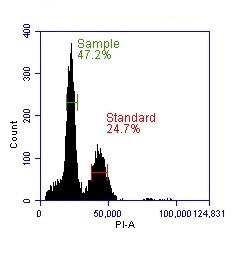


**Supplementary Figure S1.** Flow cytometry determination of the nuclear DNA content of diploid *P. granatum*. Chicken erythrocyte nuclei were used as a reference standard. The plot shows the relative DNA staining of nuclei with propidium iodide (PI).


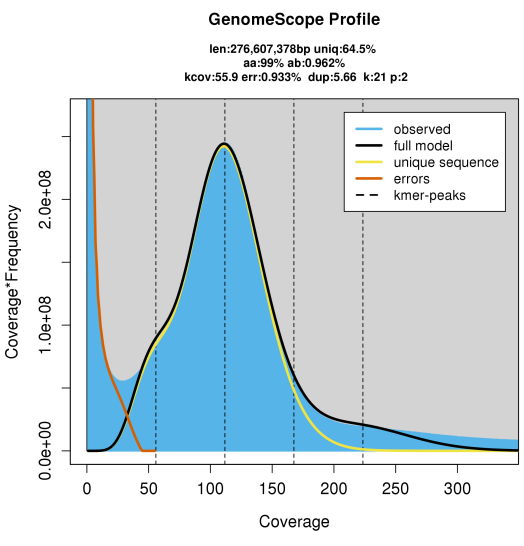


**Supplementary Figure S2.** GenomeScope k-mer profile plot for the genome of *P. granatum*, based on 21-mers in Illumina reads. The observed k-mer frequency distribution is depicted in blue, whereas the GenomeScope fit model is shown as a black line. The unique and putative error k-mer distributions are plotted in yellow and red, respectively, and the predicted assembly size is 276.60 Mb.

.
