# Supplementary figures and images for: Hybrid Assembly and Annotation of the Genome of the Indian Punica granatum, a Superfood
Source: Front Genet. 2022 May 11;13:786825. doi: 10.3389/fgene.2022.786825 (PMC9130716; doi:10.3389/fgene.2022.786825)

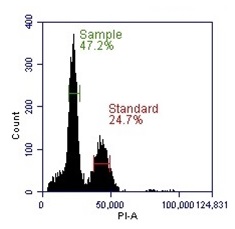

Supplement: Supplementary file 4 [file Image1.jpeg]

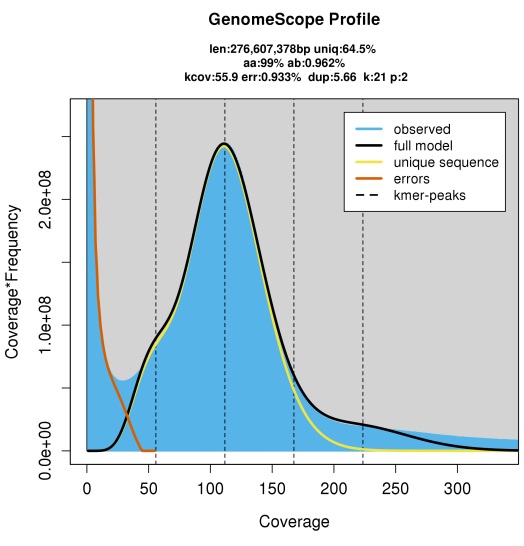

Supplement: Supplementary file 5 [file Image2.jpeg]
