# Supplementary material for: Hybrid Assembly and Annotation of the Genome of the Indian Punica granatum, a Superfood
Source: Front Genet. 2022 May 11;13:786825. doi: 10.3389/fgene.2022.786825 (PMC9130716; doi:10.3389/fgene.2022.786825)
Supplement: Supplementary file 6 [file Table5.docx]

|  |  |  |  |
| --- | --- | --- | --- |
| **Type** | **% of genome** | | |
|  | Dabenzi (China Cultivar) | Thaishanhong (China Cultivar) | Bhagwa (Indian) |
| **Transposable elements** | 46.1 | 51.2 | 26.68 |
| **Class I Retrotransposons** | 40.5 | 35.32 | 13.69 |
| **LTR Retrotransposons** | 18.9 | 0.22 | 11.43 |
| ***Gypsy*** | 9.8 | 11.55 | 0.69 |
| ***Copia*** | 4.8 | 5.8 | 1.54 |
| **Class II Transposons** | 3.4 | 6.35 | 11.23 |
| **DNA transposons** | 2.1 | 6.35 | 11.23 |
| **Unclassified** | 2.1 | 9.16 | 1.76 |

**Supplementary Table S5.** Length statistics of classified transposable elements in different cultivars of *P. granatum*
