# Supplementary material for: Hybrid Assembly and Annotation of the Genome of the Indian Punica granatum, a Superfood
Source: Front Genet. 2022 May 11;13:786825. doi: 10.3389/fgene.2022.786825 (PMC9130716; doi:10.3389/fgene.2022.786825)
Supplement: Supplementary file 8 [file Table2.doc]

Supplementary Material


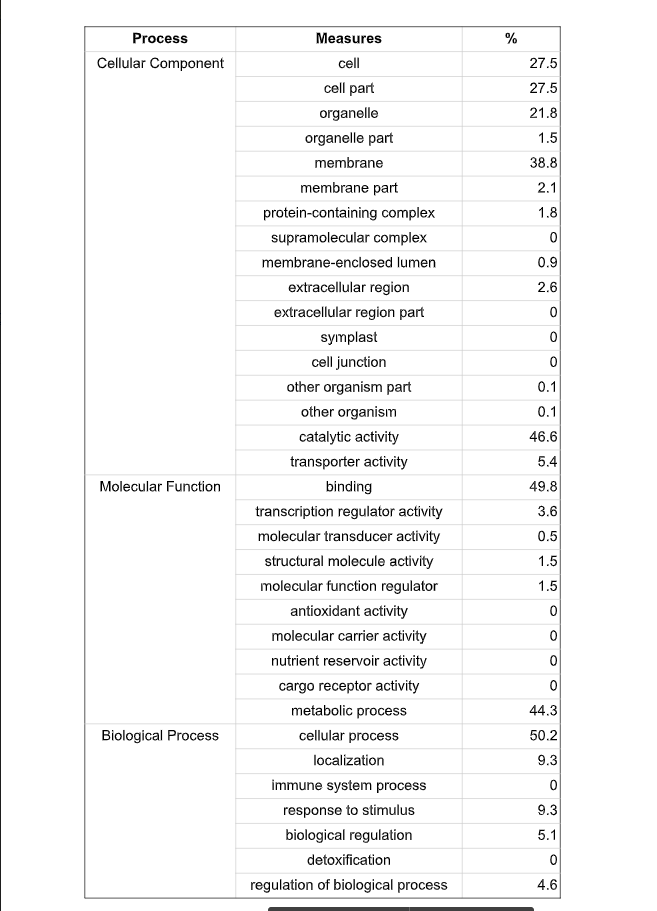


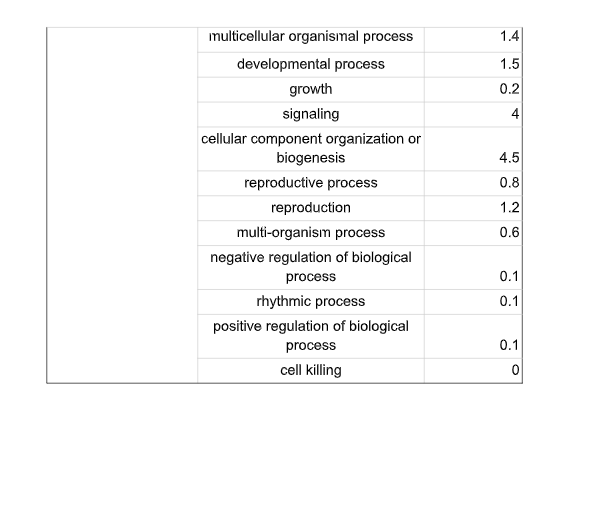


**Supplementary Table S2: Gene annotation of the functional classes in each of the three broad categories- Biological process (BP), Cellular components (CC), Molecular function (MF)**
